# TPPS_4_—Sensitized Photooxidation of Micropollutants—Singlet Molecular Oxygen Kinetic Study

**DOI:** 10.3390/molecules27165260

**Published:** 2022-08-17

**Authors:** Marta Gmurek

**Affiliations:** Molecular Engineering Department, Lodz University of Technology, Wolczanska 213, 90-924 Lodz, Poland; marta.gmurek@p.lodz.pl

**Keywords:** singlet oxygen, reaction rate constants, photooxidation, photosensitizer, kinetic

## Abstract

Visible light-sensitized oxidation of micropollutants (MPs) in the presence of meso-tetrakis(4-sulfonatophenyl)porphyrin photosensitizers was studied. In order to explore the role of type I (ROS generation) or type II (singlet oxygen) photooxidation, radical scavengers were used to obtain insight into the mechanism of photodegradation. It was revealed that singlet oxygen is the main ROS taking part in TPPS_4_- sensitized photooxidation of micropollutants. The interaction of MPs with ^1^O_2_ in deuterium oxide (D_2_O) was investigated by measuring the phosphorescence lifetime of ^1^O_2_. The rate constant (kq) for the total (physical and chemical) quenching of ^1^O_2_ by MPs was determined in a D_2_O buffer (pD 7, 9 and 10.8). The rate constants of singlet oxygen quenching and reaction with MPs were determined, and the rate constant of excited TPPS_4_ quenching by MPs was also estimated.

## 1. Introduction

The importance of singlet oxygen reaction has been recognized in many areas, including medicine, biochemistry, organic chemistry, food chemistry, and environmental engineering. Molecular oxygen possesses two unpaired electrons with parallel-directed spins in its basic state (Figure S1). Due to spin, ground-state oxygen is a triplet molecule, denoted as ^3^O_2_. The supply of energy greater than 94.2 kJ/mol to the oxygen molecule causes the rearrangement of electrons, which leads to their pairing, and the resultant spin of the molecule is zero. Changing the electronic configuration causes the singlet state ^1^O_2_ to be reached. Two different singlet oxygen forms are recognized (Figure S1), sigma singlet oxygen (^1^Σg^+^O_2_) (157 kJ/mol, 13,121 1/cm), and ^1^ΔgO_2_ delta singlet oxygen (94.2 kJ/mol, 7882 1/cm) [1]. However, ^1^Σg^+^O_2_ is a molecule that is quickly deactivated to the more stable form delta. Singlet oxygen is not a free radical; it reacts with non-reactive singlet molecules that have double bonds [2]. Chemical, enzymatic, and photochemical reactions can lead to singlet oxygen generation; however, photosensitization (photooxidation) is the most successful due to the applicability of readily available oxygen and visible light for the reaction. Several methods for identifying the type of photosensitized oxidation and, hence, singlet oxygen can be used. The first strategy is to study the effects of additives on light-initiated photochemical processes (e.g., adding sodium azide, ascorbic acid, or tryptophan to the reaction system should stop the reaction). The second method is based on the determination of known reaction products with ^1^O_2_ with selected compounds: tryptophan bleaching reactions [3]; the formation of nitroxyl radicals in reactions with sterically shielded amines—detection employing electronic paramagnetic resonance (EPR) [4]; the disappearance of disodium salt of 9,10-anthracenedipropionic acid (ADPA) measured spectrophotometrically at a wavelength equal to 400 nm [5]; and secondary RNO (p-nitro-NN-dimethylaniline) bleaching activated by reactions of singlet oxygen with histidine or imidazole [6]. A third approach is based on the direct detection of reactive species formed by the action of an excited sensitizer, e.g., direct detection of singlet oxygen. The deactivation of a single ^1^O_2_ molecule to the ground state is accompanied by the emission of electromagnetic radiation (Equation (1)), while two simultaneous electronic transitions (at 634 nm and 703 nm) can be detected during the collision of two ^1^O_2_ molecules (Equation (2)). The measurement of ^1^O_2_ phosphorescence (~1270 nm) is a specific method for its detection. Thus, the singlet oxygen emission band can be considered a kind of singlet oxygen “fingerprint,” since no other molecule shows luminescence in this range. This is probably because only a few molecules have such low values of energy states of excited molecules with transition energies similar to oxygen. The detection of singlet oxygen by phosphorescence can be carried out using time-resolved techniques or by the method of stationary emission measurement. In the time-resolved method, the photosensitizer is usually excited by a pulsed laser. The stationary method is generally used to determine the presence of singlet oxygen, while the time-resolved method can additionally determine the singlet oxygen lifetime (τ∆).

(1) O12→O32+hv1270 nm

(2) 2O12→2O32+hv1634 nm+hv2703 nm

**Figure 1:**
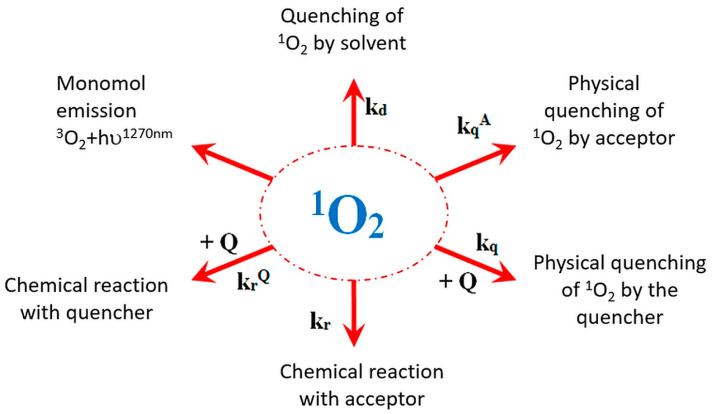
The processes of quenching to which the singlet oxygen formed as a result of photosensitization may undergo, where A—pollutant and Q—quencher, additionally in the solution, k_r_—rate constant for the chemical reaction of singlet oxygen, k_d_—rate constant of singlet oxygen decay in water, k_q_—rate constant for physical quenching of singlet oxygen.

In the gas phase, the lifetime of singlet oxygen 1Δg oscillates within 65 min, while the lifetime of ^1^Σg^+^ is only about 7 s [7]. In aqueous solutions (H_2_O), singlet oxygen lifetime is even lower (^1^Σg^+^ ~10–12 s, while ^1^ΔgO_2_ is 3.3 μs) [8]. Understanding the possibility of ^1^O_2_ quenching processes (Figure 1) allows for determining the reaction mechanism and processes competing with photochemical reactions. In the liquid phase, the dominant process of singlet oxygen deactivation is physical quenching related to the structure of the solvent (Table S1). As a result of specific interactions with solvent hydrogen atoms, ^1^ΔgO_2_ returns to the ground state in a non-radiative way. The solvent acts as an energy acceptor in collisions with singlet oxygen. Thus, the phenomenon of the physical quenching of ^1^O_2_ by the solvent is always present during the conducted reactions, and, therefore, the value of the rate constants of the physical quenching by the solvent (k_d_) is always included in the kinetic calculations.

Singlet oxygen can also interact with other compounds in the reaction mixture undergoing many quenching processes, which affect the efficiency of ^1^O_2_ formation (Figure 1). The interaction of ^1^O_2_ with molecules by transferring the excitation energy resulted in returning to the ground triplet state. It is expected that ^1^O_2_ enters into chemical reactions with molecules, and the quencher reacts with singlet oxygen to give the reaction product (chemical quenching, Equation (3)). During physical quenching, only singlet oxygen is deactivated without the consumption of molecular oxygen and product formation (Equation (4)).

(3) Q+O12→ kr P 

(4)  A/Q+O12→ kq O32+A/Q

Singlet oxygen, as a strong electrophile, readily reacts with electron pairs held by heteroatom centers. Many physical singlet oxygen quenchers are acceptors, including amines, phenols, thiols, benzene derivatives, organic transition metal complexes, inorganic anions (azides, iodides, bromides), and 1,4-diazobicyclo [2.2.2] octane (DABCO). In biological systems, β-carotene and lycopene are most commonly used as physical quenchers for singlet oxygen [9,10]. As chemical quenchers, MPs are included. The singlet oxygen quench rate constants with physical and chemical quenchers are shown in Table S2.

One of the key factors of ^1^O_2_ reactivity with MPs is the effect of pH. The change in reaction pH will affect dissociation of the MPs, which is given by the dissociation constant (pKa). It is known that when pH < pKa, MPs are predominantly in their undissociated forms, while at pH > pKa, dissociated forms are dominating. Similarly, at pH below pKa, the phenols exist in protonated form, while at pH above pKa they predominantly dissociate to phenolate ions [11]. Moreover, ^1^O_2_ reacts most easily with compounds in an anionic form Miller, 2005 [12]. This is probably associated with a single electron transfer reaction on the dissociated OH functional group and at the same time ^1^O_2_ addition to the benzene ring [11,13,14]. While phenolic compounds undergo only ^1^O_2_ addition [13].

The present investigation deals with the kinetics of the oxidation reactions of three micropollutants (butylparaben, benzylparaben and 2-chlorophenol) photosensitized by TPPS_4_ in aqueous solutions. The effect of ROS on reaction is discussed based on scavenger experiments with NaN_3_, SOD and *t*-BuOH. The main role of singlet oxygen was established by the application of time-resolved spectroscopy. The rate constants for singlet oxygen quenching by MPs were determined.

## 2. Results

It is known that various reactive oxygen species can be produced during photosensitized oxidation reactions. According to the Type I mechanism, hydroxyl radicals (^●^OH) or superoxide anion radicals (O_2_^●−^) can be generated. The Type II photosensitized oxidation mechanism produces singlet oxygen (^1^O_2_) [14]. To establish the reaction mechanism with the selected micropollutants, scavengers reacting with reactive oxygen species were applied. Firstly experiments with sodium azide (NaN_3_), a physical singlet oxygen quencher, were performed. It is known that NaN_3_ deactivates ^1^O_2_ according to reaction (5) and is characterized by a high ^1^O_2_ quenching constant [15].

(5) N3−+O12 →kq=2.0×109dm3/mol sN3•+O32  

Secondly, the involvement of ^●^OH radical was investigated. For this purpose, the possibility of ^●^OH scavenging by *tert*-butanol (*t* BuOH) according to reaction 6 was examined.

(6) O●H+CCH3OH→k=6.0×108dm3/mol sH2O+C●H2(CH3)2COH

Additionally, it was also examined whether a superoxide anion radical was present in the solution. It is known that O_2_^●−^ in the reaction of disproportionation catalyzed by superoxide dismutase (SOD) is reduced to hydrogen peroxide and oxidized to molecular oxygen according to reaction 7 [16,17]:

(7) O2● −+O2● −+2H+→SOD,   k=2.0×109dm3/molsH2O2+O32

**Figure 2:**
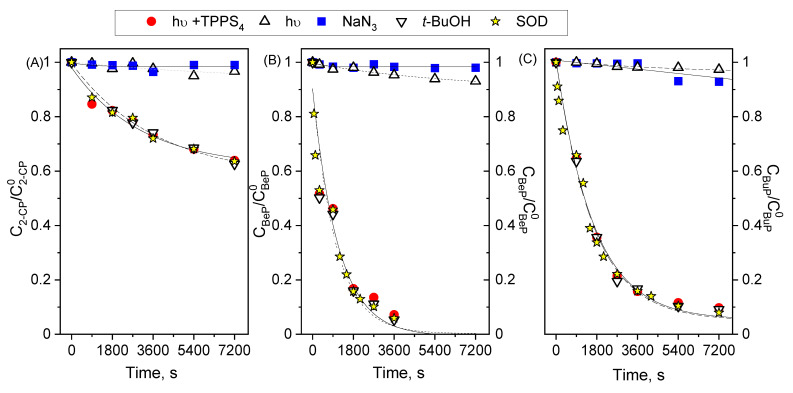
Influence of ROS scavengers on photosensitized oxidation of (**A**). 2-CP (pH = 7), (**B**). BeP (pH = 10.8); (**C**). BuP (pH = 10.8); C^2−CP^_0_ = 1 × 10^−4^ mol/dm^3^, C^BeP^_0_ = 8 × 10^−5^ mol/dm^3^, C^BP^_0_ = 8 × 10^−5^ mol/dm^3^, C^NaN3^ = 2 × 10^−2^ mol/dm^3^, C^t-BuOH^ = 1 × 10^−1^ mol/dm^3^, *E*_0_ = 3.24 × 10^−4^ einstein/s dm^3^.

As shown in Figure 2, it can be concluded that the application of NaN_3_ inhibited the decay of the reaction in all cases. The addition of t-BuOH and SOD did not affect the degradation rate of BuP, BeP, and 2-CP. The experiments with t-BuOH and SOD excluded the radical mechanism, showing that the reaction solution lacked ^●^OH and O_2_^●−^. On the other hand, the presence of NaN_3_ in the solution confirmed the oxidizing effect of singlet oxygen in relation to the selected micropollutants. The NaN_3_ presence inhibited the reaction to the level of direct photolysis of MPS (Figure 2). Based on the above experiments, it can be concluded that the decay of MPs concentration occurs as a result of the action of singlet oxygen (TYPE II) and, to a small extent (that can be neglected), photolysis.

### 2.1. Time Resolve Spectroscopy Measurements

Using the Stern–Volmer equation, the values of the rate constants of the physical and chemical quenching of ^1^O_2_ by MPs (k_t_—Equation (8)), the rate constant of the physical quenching of the triplet state of the ^3^TPPS_4_ by molecular oxygen (k_q_^3O2^—Equation (9)), and the rate constants of the physical quenching of the ^3^TPPS_4_ by MPs (k_q_^MPs^ Equation (10)) were determined.

(8) τO12−1=τ0O12−1+ktMPsCMPs0

(9) τT3PPS4−1=τ0T3PPS4−1+kqO32CO320

(10) τT3PPS4−1=τ0T3PPS4−1+kqMPsCO320

where: *τ*—phosphorescence lifetime without quencher, *τ*_0_—lifetime of phosphorescence with a quencher, and k_q_—quenching constant.

**Figure 3:**
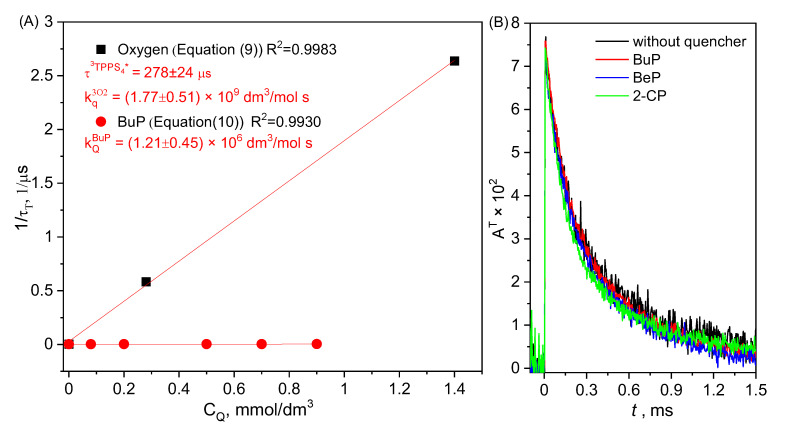
(**A**) Quenching rate constant ^3^TPPS_4_ via molecular oxygen and butylparaben, (**B**) signal of ^3^TPPS_4_ with and without MPs in alkaline solution.

As mentioned above, one of the most important quenchers of the triplet form of photosensitizer is molecular oxygen. To determine the k_q_^3O2^ constant quenching rate, experiments were carried out in D_2_O under conditions of different oxygen concentrations. The concentration of dissolved oxygen in the water was respectively 1.4 mmol/dm^3^ in a solution saturated with oxygen, 0.28 mmol/dm^3^ in a solution saturated with air, and 0 mmol/dm^3^ in a solution saturated with argon. The quenching rate constant of the triplet form TPPS_4_ by molecular oxygen was determined as k_q_^3O2^ = (1.77 ± 0.05) × 10^9^ dm^3^/mol s (Figure 3A). This value is consistent with that reported in the literature: 1.8 × 10^9^ dm^3^/mol s [18], (1.5 ± 0.1) × 10^9^ dm^3^/mol s [19] and (2.2 ± 0.1) × 10^9^ dm^3^/mol s [20].

The influence of BuP, BeP, and 2-CP on triplet TPPS_4_ was also investigated. The experiments were conducted at pD 7 and 10.8. It was found that in a neutral environment, only the presence of BuP caused quenching of TPPS_4_ triplet form with the reaction rate constant k_q_^BuP(7)^ = (1.2 ± 0.5) × 10^6^ dm^3^/mol s, while, in the alkaline environment, no significant differences were observed in the presence of BuP, BeP, and 2-CP compared to the phosphorescence signal of the excited form of TPPS_4_ without any quencher (Figure 3B). It can therefore be assumed that the impact of the quenching is negligible.

**Figure 4:**
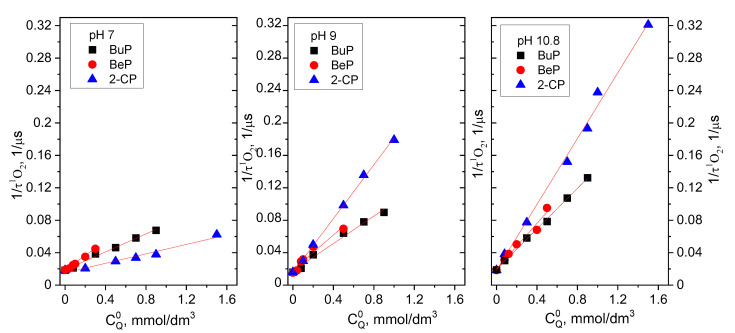
Determination of the bimolecular quenching rate constants k_q_^t^ of quenching of ^1^O_2_ in different pD by MPs. The slope of the plot of the inverse singlet lifetime (monitored at 1270 nm) vs. the quencher concentration.

The singlet oxygen quenching process by the MPs was performed in deuterium water at various pD values of 7, 9, and 10.8. It has been already discussed that the lifetime of singlet oxygen is highly solvent-dependent. In D_2_O, ^1^O_2_ has a lifetime of about 12 to 20 times higher than in H_2_O [15,21]. In this study, the lifetime of ^1^O_2_ in D_2_O was equal to 52 ± 3 μs. The k_t_ values for each compound were determined using the Stern–Volmer Equation (8). The results are presented in Figure 4 and Table 1. As can be seen, the rate constant is dependent on the pD of the reaction medium. The highest values are observed in an alkaline environment and decrease with increasing acidity. This is in agreement with higher ^1^O_2_ reactivity toward phenolate rather than phenol. Electron transfer between ^1^O_2_ and the dissociated OH functional group requires much less energy than the transfer between the benzene ring and ^1^O_2_ [13], resulting in a faster reaction during photooxidation of phenolates.

### 2.2. Kinetic Models for Photooxidation 

The following reactions take place in the photodegradation process of micropollutants with the use of TPPS_4_ as. a photosensitizer. The reaction is initiated by the absorption of visible light by the photosensitizer, which transfers to the excited triplet state (^3^TPPS_4_*) with a quite high quantum yield (Equation (11), Table 2). ^3^TPPS_4_* has a long enough lifetime to participate in many chemical processes, or may go back to the ground state as it undergoes phosphorescence. If oxygen is available in an aqueous solution, ^3^TPPS_4_* can quench by either chemically producing ^1^O_2_ or physically dissipating energy. Similarly, the micropollutant can react with ^3^TPPS_4_* via physical quenching. The excited form of porphyrin in the triplet state can react with TPPS_4_ in the ground state (Equation (14)) or directly return to the ground state (Equation (15)). ^3^TPPS_4_* can also be physically quenched by MPs to revert to its ground state (Equation (18)) or it can also be physically quenched by molecular oxygen (Equation (16)). The possible reaction pathway is presented below:

(11) TPPS4+hυ→                T1PPS4*→    ϕT  Ea         T3PPS4*

(12) T3PPS4*+O32→  kΔO2    O12+TPPS4

(13) EDCs+O12→  krEDCs    photodegradation product

(14) T3PPS4*→  kD         TPPS4

(15) T3PPS4*+TPPS4→       kqTPPS4         (TPPS4)•–+(TPPS4)•+ 

(16) T3PPS4*+O32→  kq3O2    product

(17) TPPS4+O12→  krTPPS4    photodegradation product

(18) T3PPS4*+MPs →  kqMPs    TPPS4+MPs

(19) T3PPS4*+MPs →  krTPPS4MPs    product

(20) O12→  kd    O32

(21) EDCs+O12→  kqMPs    EDCs+O32

As a result of Equation (12), singlet oxygen is formed, which can be physically quenched by MPs (Equation (21)) or can be returned directly to the ground state (Equation (20)).

^3^TPPS_4_* reacts with oxygen, initiating the photodegradation reaction of MPs. This photosensitizer in the excited state may also directly affect the disappearance of MPs according to Equations (18) and (19). However, in accordance with results obtained with NaN_3_, the reaction described by Equation (19) does not occur, which is consistent with the literature [12]. Based on the performed experiments and considering photolysis, it can be assumed that the only pathway to decompose BuP, BeP, and 2-CP is a process based on the mechanism of energy transfer to oxygen.

According to the above-mentioned reaction mechanism, it can be assumed that photolysis has little effect on the decomposition of MPs, considering that there is only a physical reaction with the photosensitizer in the triplet state (Equation (18)). In addition, following the experimental results with ROS scavengers, the rate of decay of selected MPs can be determined from the equation:

(22) r=−dCMPsdt=krMPs CMP CO12+kr′MPs− CMP− CO12

where k_r_ is the reaction constant of the MP with singlet oxygen (chemical quenching) and k_r_ ‘ is the rate constant of the degradation reaction in the dissociated MPs form with singlet oxygen.

In a neutral environment (pH 7), when MPs are present practically only in undissociated form (α = 0.0544, α = 0.0568, and α = 0.0262 for BuP, BeP, and 2CP, respectively), the second part of the equation can be omitted. However, in an alkaline solution (pH 10.8), the ionic form of BuP, BeP, and 2-CP is predominantly (α > 99%), therefore the first part of the reaction can be neglected.

Applying an approximation of the steady-state concentrations of ^1^O_2_ and ^3^TPPS_4_*, the following equation is presented:

(23) CO12=kΔO2 CT3PPS4* CO32kd+kqMP+krMP CMP+krTPPS4 CTPPS4

(24) CT3PPS4*=Ea ϕTkD+kqTPPS4 CTPPS4+kqO2 CO32+kqMP CMP 

where E_a_ is the intensity of the absorbed radiation, i.e., the number of absorbed photons per unit of time.

Low values of k_D_ [22] and k_q_^MP^ (which were determined experimentally) and the applied low concentration of TPPS_4_ (2 × 10^−5^ mol/dm^3^) allow the omission of their values in Equations (23) and (24). The final equation for ^1^O_2_ concentration is equal to:

(25) CO12=kΔO2 Ea ϕT CO32(kd+ktMP CMP) (kQO2 CO32+kQMP CMP)

where $k_{t}^{MP}=k_{q}^{MP}+k_{r}^{MP}$.

Considering that Equation (25) is a part of Equation (22), the reaction rate of photosensitization of the undissociated (pH 7) MP form and the dissociated MP form (pH 10.8) is given by Equation (26).

(26) r=−dCMPdt=krMP CMP Ea ϕΔ(kd+ktMP CMP)1+kQMP CMP kQO2 CO32

where $\phi_{\Delta }=\phi_{T\;}\frac{k_{\Delta }^{O_{2}}}{k_{Q}^{O_{2}}}$.

For experimental data obtained at pH 7 and 10.8, the application of Equation (26), during the beginning phase of the reaction, allowed for the estimation of the unknown kinetic parameters: $k_{r}^{\mathrm{MP}}$, $k_{t}^{\mathrm{MP}}$, and $k_{q}^{\mathrm{MP}}$. The other values were taken from the literature (Table 2). By differentiating the MP decay curve that matched experimental sites with a correlation factor greater than 0.97, the initial reaction rates were determined. In order to account for the rate of direct photolysis, the values of r_0_ were modified.

It should be highlighted that when the excited photosensitizer is weakly quenched by the micropollutant in comparison to triplet oxygen quenching, or when the quotient in the denominator is significantly lower than 1, Equation (26) can be simplified (Equation (27)). This hypothesis was typically used to characterize the kinetics of sensitized oxidation [12,14,24,25].

(27) $$r=-\frac{dC_{MP}}{dt}=\frac{k_{r}^{MP}\;C_{MP}\;E_{a}^{}\;\phi_{\Delta }}{\left(k_{d}+k_{t}^{MP}\;C_{MP}\right)}$$

Two methods were adopted in the modelling process:

**Method 1**—consisted of determining the values of the constants $k_{t}^{MP}$ and $k_{q}^{MP}$ determined by time-resolved spectroscopy (TRS), while the constant $k_{r}^{MP}$ was determined by fitting the model to the experimental data,

**Method 2**—consisted of determining all three reaction rate constants from the fit, and the parameters determined by the TRS method were used as starting values.

It should be remembered that in the case of the approach using method 1, the constants $k_{t}^{MP}$ and $k_{q}^{MP}$ were determined by time-resolved spectroscopy using D_2_O as a solvent. The application of method 1 is therefore only comparative and does not describe the kinetics of the process taking place in an aqueous solution, where the effect of ^1^O_2_ quenching by the solvent (H_2_O (k_d_ = 2.4 × 10^5^ 1/s),) is much higher than in D_2_O (k_d_ = 1.9 × 10^4^ 1/s). Therefore, the use of method 2 for models (simplified (Equation (27) and not simplified (Equation (26)) gives estimated values of the kinetic constants of the ongoing process. It should also be considered that the measurement with the TRS technique is performed within a very short period of time from the activation of the photosensitizer (μs), while experimental research was performed for 2 h. The first sample was collected within 5 or 15 min of the process, therefore the processes of quenching the triplet form of the photosensitizer or physical quenching of singlet oxygen by MP may be much more noticeable. The reason for the inhibition of the reaction rate of the photosensitized oxidation process may therefore be either the formation of photosensitizer-compound complexes or the formation of paraben aggregates at higher concentrations of compounds. In concentrations above 1 × 10^−4^ mol/dm^3^ at pH < 9, parabens tend to form aggregates [26], therefore a decrease in the reaction rate of the process is observed in a neutral environment.

**Figure 5:**
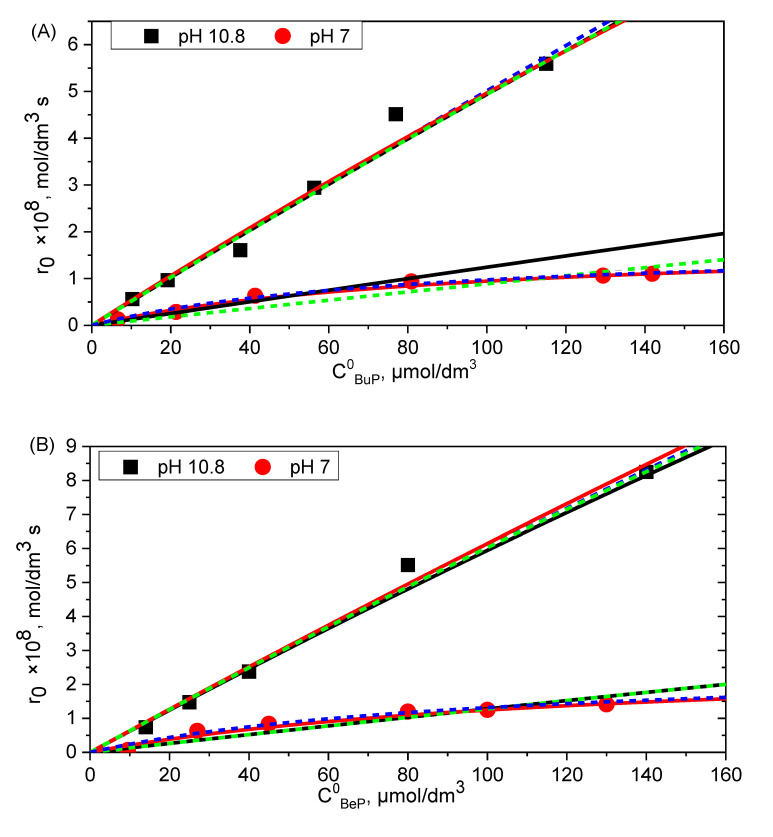

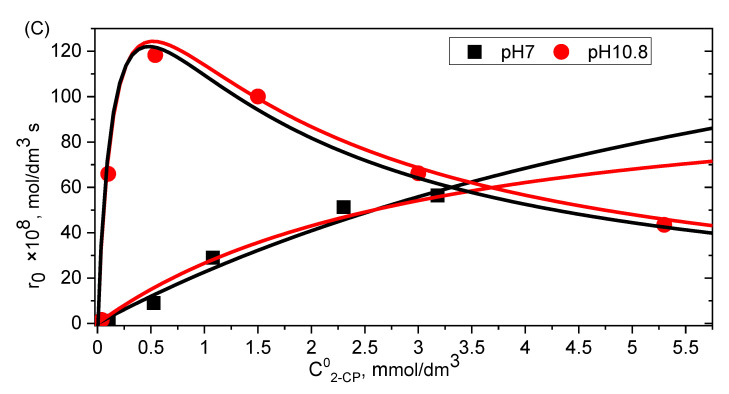
Dependence of reaction rate on initial BuP (**A**), BeP (**B**), and 2-CP (**C**) concentration simulated according to Equation (26) and Equation (27) for determining rate constants under aerated solution (0.28 mM O_2_) at the neutral and alkaline solution. Points represent experimentally determined initial reaction rates (corrected due to direct photolysis). (▬ Equation (26), method 1; ▬ Equation (26), method 2; ▪▪▪▪ Equation (27), method 1; ▪▪▪▪ Equation (27), method 2), *E*_0_ = 3.24 × 10^−4^ einstein s^−1^ dm^−3^.

These two approaches allowed for the application of a kinetic model for determining the reaction rate constants. The obtained results are presented in Figure 5, while the obtained values of the kinetic constants are presented in Table 3, Table 4 and Table 5.

## 3. Discussion

As can be seen, the kinetic models described by Equations (26) and (27) describe the experimental data well for butylparaben and benzylparaben. The obtained kinetic constants determined for both models are similar. For 2-chlorophenol, the application of the simplified model (27) was impossible due to the decrease in the initial reaction rates at high concentrations of 2-CP, observed in both reaction media. The same effect of the initial concentration on the rate constant of the reaction of singlet oxygen with 4-chlorophenol was observed by Tratynyek and Holhne [11].

It is impossible to compare the determined values of the reaction rate constants because there are no published data on the kinetics of BuP and BeP degradation by singlet oxygen. However, other studies using substances with a similar structure have been examined under photosensitized oxidation (Table 6). The kinetic constants for the photosensitized oxidation of 2-chlorophenol have already been thoroughly studied and are accessible in the literature (Table 6). The values of the rate constant of the dissociated and undissociated forms of 2-CP with singlet oxygen agree with those published for 2-CP, 3-CP, and 4-CP.

## 4. Materials and Methods

*Meso*-tetrakis(4-sulfonatophenyl)porphyrin (TPPS_4_, Fluka), *n*-butylparaben, benzylparaben, and 2-chlorophenol (>99%, BP and BeP; 2-CP 98% (purchased from Fluka, Steinheim, Germany) were used as received.

Experiments were performed in a series of five flat plate reactors made from optical glass symmetrically positioned around the lamp. The distance between the inner walls of the plate reactor was 0.3 cm, and the capacity of the reactor amounts to 0.01 dm^3^. The photon flux entering the reaction space was measured using Reinecke’s actinometer for wavelengths ranging from 310 to 770 nm [15,27,28]. The experiments were carried out in phosphate buffer solutions (NaH_2_PO_4_–K_2_HPO_4_; p.a. POCH S.A., Gliwice, Poland) for pH 5–8 and carbonate buffer (Na_2_CO_3_–NaHCO_3_, p.a. POCH S.A., Gliwice, Poland) for pH 9–10.8. All solutions were prepared in distilled water and additionally treated in Millipore Milli-Q Plus system. The reaction solution was aerated by gas bubbling (air, oxygen, or nitrogen). The samples for analysis were taken regularly in time. When reaction underwent at pH above 8, the samples were acidified with 0.5 M sulfuric acid (p.a. POCH S.A., Gliwice, Poland). The MPs decay was monitored by HPLC apparatus (Waters Ltd., Watford, UK) with a UV diode array detector. The chromatograph was equipped with a Nova-Pak 150/C18 column (Waters Ltd., Watford, UK). A mixture MeOH-acidified water (0.1% H_3_PO_4_) was used as a mobile phase. The analytical procedure can be found in [15,27,28].

Time-resolved measurements were performed by an FL 3002 dye laser (*λ*_exc_ = 425 nm for TPPS_4_, and output energy 0.1–3 mJ/pulse, pulse width ~28 ns) was pumped by a COMPEX102 XeCl excimer laser (both Lambda Physik, Fort Lauderdale, FL, USA). The time profiles of the triplet states of photosensitizer were probed using their absorption at 460 nm with a 150 W Xe lamp with a pulse unit on an LKS20 laser kinetic spectrometer (Applied Photophysics, Leatherhead, UK). Time-resolved near-infrared phosphorescence at 1270 nm was used to monitor the production of ^1^O_2_. After passing through a 1270 nm band-pass filter (Laser Components, Olching, Germany), the time-resolved phosphorescence was observed with a Ge diode (Judson J16-8SP-R05M-HS, Toledyne Judson Technologies, Montgomeryville, PA, USA). A 600 MHz oscilloscope (Agilent Infiniium, Colorado Springs, CO, USA) was used to gather the detector’s signal, and the data was then uploaded to a computer for further analysis. Individual traces were accumulated 200 times to improve the signal-to-noise statistics. The samples were exposed to air or oxygen as necessary, and argon purging was used to eliminate oxygen from the solution.

The initial reaction rates were calculated by differentiating the exponential curve that fitted experimental points (C, *t*) at a correlation factor higher than 0.97.

## 5. Conclusions

The course of the reaction was also observed in the presence of certain additives which act as scavengers of reactive oxygen species, allowing an explanation of the mechanism of the reactions taking place. The rate of photodegradation of parabens in alkaline and neutral environment increased with the increase of the initial substrate concentration, and a different relationship can be observed for 2-chlorophenol (2-CP). In a neutral environment, the increase in the photodegradation rate of butylparaben (BuP) and benzylparaben (BeP) is hyperbolic, while in an alkaline environment, it is directly proportional. There is a strong dependence on the initial substrate concentration during the degradation of 2-CP. In a neutral environment, a hyperbolic increase in the rate of the process can be observed with an increase in the initial concentration. On the other hand, in an alkaline environment, the dependence of the reaction rate on the initial concentration of 2-CP shows the existence of an optimal concentration of 2-CP (5.4 × 10^−4^ mole/dm^3^), at which the highest rate of photodegradation reaction is obtained.

The main purpose of the research was to determine the kinetic parameters of the reactions studied. Often, in works devoted to the removal of pollutants from the aquatic environment, a purely practical approach is used. As a result, the issues of reaction rate are presented marginally, and the main emphasis is on describing the efficiency of the process. Kinetic relationships are simplified and most often consist of fitting experimental data to first-order equations. The experiments presented in this paper made it possible to understand the process at the molecular level, which allowed for the determination of the reaction mechanism and the correct construction of the kinetic model. The kinetic quantities determined in this way have a specific physical meaning. Moreover, the kinetic equations derived from the reaction mechanism are valid over a wider range of variables as compared to the macrokinetic equations.

## Figures and Tables

**Table 1:** Bimolecular quenching rate constants (physical and chemical) k_q_^t^ of quenching of ^1^O_2_ in different pD by MPs.

**pH 7**			
k_t,_ dm^3^/mol s	BuP	BEP	2-CP
	(5.06 ± 0.2) × 10^7^	(8.86 ± 0.3) × 10^7^	(2.92 ± 0.3) × 10^7^
**pH 9**			
k_t,_ dm^3^/mol s	BuP	BEP	2-CP
	(8.45 ± 0.5) × 10^7^	(1.02 ± 0.04) × 10^8^	(1.66 ± 0.03) × 10^8^
**pH 10.8**			
k_t,_ dm^3^/mol s	BuP	BEP	2-CP
	(1.15 ± 0.04) × 10^8^	(1.21 ± 0.06) × 10^8^	(2.03 ± 0.07) × 10^8^

**Table 2:** The rate constants of the components of the reactions taking place during photooxidation of a solution containing TPPS_4_ in the presence of oxygen.

Constant	φ_T_	φ_Δ_	k_D_ 1/s	$k_{Q}^{O_{2}}\;{\mathrm{dm}}^{3}/\mathrm{mol}\;s$	kΔO32dm3/mol s	k_d_ 1/s	k_r_^TPPS4^ dm^3^/mol s
Value	0.78 [23]	0.62 [18]	2.8 × 10^3^ [22]	1.77 × 10^9^ [this study]	1.46 × 10^9 a^	2.4 × 10^5^ [21]	<10^8^ [14]

^a^ Calculated $\phi_{\Delta }=\phi_{T\;}\frac{k_{\Delta }^{O_{2}}}{k_{Q}^{O_{2}}}$.

**Table 3:** Determined kinetic rate constant of BuP reaction with singlet oxygen and excited ^3^TPPS_4_* with the application of Equation (26) and Equation (27) with two approaches.

Rate constant, dm^3^/mol s	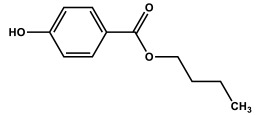		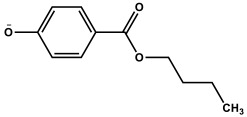	
	pH = 7		pH = 10.8	
	**Equation (26)**			
	Method 1	Method 2	Method 1	Method 2
$k_{r}^{BuP}$	(1.25 ± 0.5) × 10^6^	(1.9 ± 0.1) × 10^6^	(5.1 ± 0.4) × 10^6^	(5.3 ± 0.6) × 10^6^
$k_{t}^{BuP}$	(5.1 ± 0.2) × 10^7^	(2.5 ± 0.5) × 10^9^	(1.1 ± 0.04) × 10^8^	(2.0 ± 0.2) × 10^8^
$k_{q}^{BuP}$	(1.2 ± 0.5) × 10^6^	(2.12 ± 0.9) × 10^6^	no quenching	(1.01 ± 0.7) × 10^6^
Rate constant, dm^3^/mol s ^1^	**Equation (27)**			
	pH = 7		pH = 10.8	
	Method 1	Method 2	Method 1	Method 2
$k_{r}^{BuP}$	(8.96 ± 0.8) × 10^5^	(2.14 ± 0.3) × 10^6^	(5.1 ± 0.4) × 10^6^	(5.1 ± 0.09) × 10^6^
$k_{t}^{BuP}$	(5.1 ± 0.2) × 10^7^	(2.95 ± 0.8) × 10^9^	(1.1 ± 0.04) × 10^8^	(7.2 ± 0.8) × 10^7^

**Table 4:** Determined kinetic rate constant of BeP reaction with singlet oxygen and excited ^3^TPPS_4_* with the application of Equation (26) and Equation (27) with two approaches.

Rate constant, dm^3^/mol s	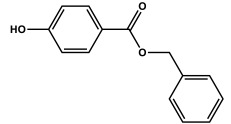		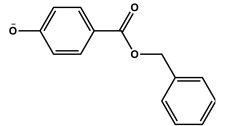	
	pH = 7		pH = 10.8	
	**Equation (26)**			
	Method 1	Method 2	Method 1	Method 2
$k_{r}^{BeP}$	(1.31 ± 0.1) × 10^6^	(2.2 ± 0.4) × 10^6^	(6.2 ± 0.4) × 10^6^	(6.3 ± 0.07) × 10^6^
$k_{t}^{BeP}$	(8.8 ± 0.3) × 10^7^	(1.9 ± 0.1) × 10^9^	(1.4 ± 0.3) × 10^8^	(1.5 ± 0.2) × 10^8^
$k_{Q}^{BeP}$	no quenching	(5.5 ± 1.1) × 10^7^	no quenching	(3.2 ± 0.4) × 10^6^
Rate constant, dm^3^/mol s ^1^	**Equation (27)**			
	pH = 7		pH = 10.8	
	Method 1	Method 2	Method 1	Method 2
$k_{r}^{BeP}$	(1.31 ± 0.1) × 10^6^	(2.6 ± 0.2) × 10^6^	(6.20 ± 0.2) × 10^6^	(6.21 ± 0.8) × 10^6^
$k_{t}^{BeP}$	(8.8 ± 0.3) × 10^7^	(2.4 ± 0.1) × 10^9^	(1.4 ± 0.3) × 10^8^	(7.9 ± 0.1) × 10^7^

**Table 5:** Determined kinetic rate constant of 2CP reaction with singlet oxygen and excited ^3^TPPS_4_* with the application of Equation (26) with two approaches.

Rate constant, dm^3^/mol s	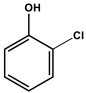		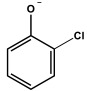	
	pH = 7		pH = 10.8	
	**Equation (26)**			
	Method 1	Method 2	Method 1	Method 2
$k_{r}^{2-CP}$	(2.5 ± 0.3) × 10^6^	(3.4 ± 0.1) × 10^6^	(1.22 ± 0.02) × 10^8^	(1.15 ± 0.3) × 10^8^
$k_{t}^{2-CP}$	(2.9 ± 0.4) × 10^7^	(6.8 ± 0.2) × 10^7^	(2.03 ± 0.2) × 10^8^	(1.88 ± 0.02) × 10^8^
$k_{Q}^{2-CP}$	no quenching	(3.0 ± 0.7) × 10^6^	(2.4 ± 0.08) × 10^9^	(2.22 ± 0.03) × 10^9^

**Table 6:** Kinetic constants for singlet oxygen with similar structure MP available in the literature (k_rArOH_/k_tArOH_—undissociated form, k_rArO_^−^/k_tArO_^−^—dissociated form).

Compound	k_rArOH_ dm^3^/mol s	k_rArO_^−^ dm^3^/mol s	k_tArOH_ dm^3^/mol s	k_tArO_^−^ dm^3^/mol s
phenol	(2.6 ± 4.0) × 10^6 (a)^	(1.55 ± 0.05) × 10^8 (a)^	-	-
4′-Hydroxy acetophenone	(1.5 ± 0.1) × 10^6 (a)^	(2.36 ± 0.01) × 10^7 (a)^	-	-
4-hydroxyphenol	(3.8 ± 5.5) × 10^7 (a)^	-	-	-
2-benzylphenol	-	4.4 × 10^7 (b)^	-	-
salicylic acid methyl ester	-	<2 × 10^6 (c)^		1.2 × 10^8 (c)^
4-chlorophenol	(6.0 ± 3.6) × 10^6 (a)^	(1.93 ± 0.04) × 10^8 (a)^	-	-
3-chlorophenol	(5.4 ± 1.0) × 10^6 (a)^	(1.6 ± 0.02) × 10^8 (a)^	-	-
2-chlorophenol	(9.2 ± 9.4) × 10^6 (d)^	(1.92 ± 0.1) × 10^8 (d)^	-	-
	-	1.7 × 10^7 (d)^	-	2.3 × 10^8 (d)^
	-	2.2 × 10^8 (e)^	-	6.8 × 10^8 (e)^
	3.8 × 10^5 (e)^	2.05 × 10^8 (e)^	1.33 × 10^6 (e)^	2.36 × 10^9 (e)^

^(a)^ [11], ^(b)^ [25] in H_2_O, ^(c)^ [24] pH = 10, ^(d)^ [25] Palumbo i in., 1990, ^(e)^ [12].

## Data Availability

Not applicable.

## Supplementary Materials

The following supporting information can be downloaded at: https://www.mdpi.com/article/10.3390/molecules27165260/s1, Figure S1. Distribution of electrons in the molecular orbitals of oxygen in the ground and singlet states. Arrows symbolize electrons, electron spins are marked with arrows; Table S1. Lifetime of ^1^ΔgO_2_ in different solvents; Table S2. Constant rates of singlet oxygen reactions with physical and chemical quenchers. References [29,30,31,32] are cited in the Supplementary Materials.

## Nomenclature/Abbreviations

^1^O_2_	singlet oxygen
BuP	butylparaben
BeP	benzylparaben
2CP	2-chlorophenol
MPs	micropollutants
E_a_	flux of absorbed photons per unit of the reaction volume
k	rate constant
k_d_	rate constant of singlet oxygen decay in water
k_q_	rate constant for physical quenching of singlet oxygen
k_r_	rate constant for the chemical reaction of singlet oxygen
$k_{r}^{TPPS_{4}}$	rate constant for chemical reaction of singlet oxygen with TPPS_4_
$k_{t}^{MPs}$	rate constant for physical and chemical decay of singlet oxygen with MP
k_D_	rate constant for triplet TPPS_4_ decay
kΔO32	rate constant for triplet TPPS_4_ reaction with oxygen which gives ^1^O_2_
$k_{Q}^{O_{2}}$	rate constant for triplet TPPS_4_ quenching by oxygen
$k_{q}^{TPPS_{4}}$	rate constant for triplet TPPS_4_ quenching by ground state TPPS_4_
$k_{q}^{MPs}$	rate constant for triplet TPPS_4_ quenching by MPs
O_2_	molecular oxygen (triplet ^3^O_2_)
r	reaction rate
TPPS_4_	*meso*-tetrakis(4-sulfonatophenyl)porphyrin
t	time
α	dissociation degree
φ	quantum yield
φ_T_	quantum yield of triplet formation
φ_Δ_	quantum yield of singlet oxygen formation
λ	wavelength
*	excited state
0	initial conditions
