# Supplementary material for: TPPS4—Sensitized Photooxidation of Micropollutants—Singlet Molecular Oxygen Kinetic Study
Source: Molecules. 2022 Aug 17;27(16):5260. doi: 10.3390/molecules27165260 (PMC9412460; doi:10.3390/molecules27165260)
Supplement: Supplementary file 1 [file molecules-27-05260-s001.zip › molecules-1840507-supplementary.pdf]

Supplementary Information

$TPPS_4$—Sensitized Photooxidation of Micropollutants—Singlet Molecular Oxygen Kinetic Study

Marta Gmurek

Molecular Engineering Department, Lodz University of Technology, Wolczanska 213, 90-924 Lodz, Poland; marta.gmurek@p.lodz.pl

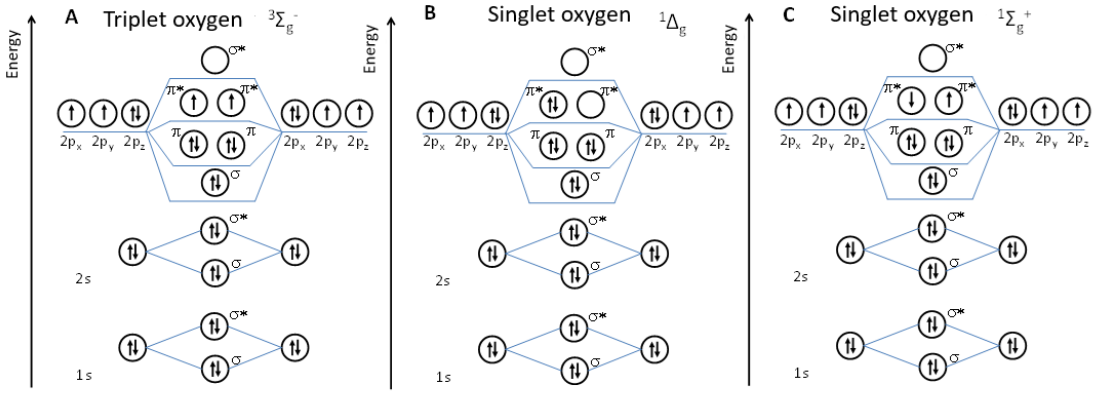

Figure S1. Distribution of electrons in the molecular orbitals of oxygen in the ground and singlet states. Arrows symbolize electrons, electron spins are marked with arrows.

Table S1. Lifetime of $^1\Delta gO_2$ in different solvents [8,15,21,29].

solvent	τ_Δ, μs	$k_d\times10^{-4}$, 1/s	solvent	τ_Δ, μs	$k_d\times10^{-4}$, 1/s
H_2O	3.3	50±20	C_2H_3N	75	3.3±0.7
D_2O	67	3.0±0.2	CH_2Cl_2	94	1.6±0.7
$C_6H_5CH_3$	30	4.0	$CHCl_3$	247	0.44±0.18
CH_3OH	10.4	14±2	CS_2	34 000	0.5±0.15
C_6H_6	31	4.2±0.9	C_6D_6	700	0.27±0.06
$C_2Cl_3F_3$	99 000	-	C_6F_6	3900	0.17±0.6

Table S2. Constant rates of singlet oxygen reactions with physical and chemical quenchers.

Quencher	Quenching type	Rate constant $M^{-1}\ s^{-1}$	Reference
NaN_3	physical	k_q= 4.78×10^8 (D_2O)	[30]
		k_q= 2×10^9 (H_2O)	[31]
		k_q= 2.8×10^9 (CH_3CN)	[30]
DABCO		k_q= 1.2×10^7 (H_2O)	[31]
imidazole		k_q= 2×10^7 (D_2O)	[32]
β-carotene		k_q= 1.4×10^{10}	[28]
likopen		k_q= 3.1×10^{10}	
Histidine	Physical and chemical	k_q= 5×10^7	[28]
1-methyl-cyclopentane	chemical	k_r= 2.1×10^5 (CH_3OH)	[29]
4-methyl-cyclohexane		k_r= 3.1×10^3 (CH_3OH)	

**3-methylstyrene**	$k_r = 5.0\times10^6$	
**2-phenylfuran**	$k_r = 9\times10^6$ (H_2O)	[11]
**2-chlorophenol anion**	$k_r = 1.9\times10^8$ (H_2O)	
